# Effect of surfactant concentration in electrolyte on the fabrication and properties of nickel-graphene nanocomposite coating synthesized by electrochemical co-deposition†

**DOI:** 10.1039/c7ra13651j

**Published:** 2018-05-31

**Authors:** Ghulam Yasin, Muhammad Arif, Muhammad Naeem Nizam, Muhammad Shakeel, Muhammad Abubaker Khan, Waheed Qamar Khan, Tahira Mehtab Hassan, Zaheer Abbas, Iman Farahbakhsh, Yu Zuo

**Affiliations:** State Key Laboratory of Chemical Resource Engineering, Beijing University of Chemical Technology Beijing 100029 P. R. China zuoy@mail.buct.edu.cn yasin@mail.buct.edu.cn; BUCT-CWRU International Joint Laboratory, State Key Laboratory of Organic-Inorganic Composites, Center for Soft Matter Science and Engineering, College of Energy, Beijing University of Chemical Technology Beijing 100029 P. R. China; College of Materials Science and Engineering, Beijing Institute of Technology Beijing 100081 P. R. China; School of Materials Science and Engineering, Beijing University of Technology Beijing 100029 P. R. China; Department of Physics, The Islamia University of Bahawalpur Bahawalpur 63100 Pakistan; Department of Engineering, Quchan Branch, Islamic Azad University Quchan Iran

## Abstract

Long-time environmental protection of metallic materials is still required in the manufacturing and engineering applications. Nickel-graphene nanocomposite coatings have been prepared on carbon steel using sodium dodecyl sulfate (SDS) as a dispersant in the electrolyte by an electrochemical co-deposition technique. In this study, the effects of surfactants on graphene dispersion, carbon content in the coatings, surface morphology, microstructures, microhardness and corrosion resistance properties of the nanocomposite coatings are explored. The results indicate that the reasonably good graphene dispersion, coarser surface morphology and reduction in grain sizes are achieved upon increasing the surfactant concentration in the electrolyte. The surfactant also influences the preferred orientation of grains during electrodeposition; the (200) plane is the preferred orientation for the nanocomposite produced with SDS in the bath electrolyte. The microhardness, adhesive strength and corrosion performance of the nickel-graphene nanocomposite coatings are found to increase with the increasing concentration of sodium dodecyl sulfate in the deposition bath. Moreover, the influencing mechanism of surfactant concentration on the properties of nanocomposite coatings has been discussed.

## Abstract 2

Long-time environmental protection of metallic materials is still required in the manufacturing and engineering applications.

## Graphical abstract

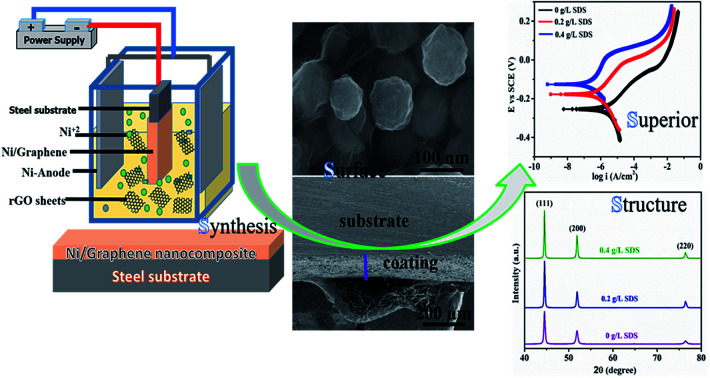

## 1. Introduction

Electrodeposition of nickel and its composites is a widely applied technique in industries to tailor the surface properties of substrate materials for the engineering applications. For several decades, various attempts have been made to achieve superior properties and better performance of coatings *via* co-deposition of different hard particles or micron fibers as a strengthening phase into a nickel metal matrix.^1–5^ A previous study has demonstrated that the characteristics of a metal matrix based composite can be improved by incorporating second-phase reinforcement particles into the host metal matrix. A variety of hard particles ,^6^ such as SiC,^7^ WC,^8^ diamond,^9^ TiO_2_,^10^ Al_2_O_3_,^11^ SiO_2_,^12^ CNTs^13^ and graphene nanosheets,^14–17^ owing to their enormous mechanical and excellent electrochemical properties^18^ can be the desired reinforcement particles to be incorporated into a metal matrix. As electrochemical co-deposition is a process largely used for the reinforcement of submicron and nanosized metallic particles, polymer additives or nonmetallic compounds in the metal matrix, it is advantageous over other techniques, for example, this process involves a simple and controllable setup, it can be performed at about room temperature, it is capable and effective to coat large, complex and irregular-shaped components and it is a cost-effective process.^19–22^ Recently, electrochemically deposited composite coatings have attracted significant attention due to their extensive properties.^23^ In fact, the morphology, structure and properties of composite coatings fabricated by the electrochemical co-deposition process are greatly affected by different deposition parameters including electrolyte composition, deposition current density, pH, additives, bath temperature and characteristics of reinforcements.^24–29^ Moreover, much effort has been devoted to study the effects of the operating parameters on the co-deposition mechanism and properties of the deposit.^30–32^

As it is well-known, the agglomeration of submicron and nanosized particles existing in the electrolyte during the deposition process is due to high ionic strength of the electrolyte and the surface free energy of particles.^33^ Moreover, poor mechanical properties of coatings are obtained due to the agglomeration of nanoparticles. Therefore, several attempts have been made to achieve a high co-deposition content and its uniform distribution. Hence, many chemical and physical methods have been performed by different researchers;^34,35^ among them, the addition of organic surfactants is an effective method to overcome the agglomeration problem in coatings.^36^

Liu *et al.*^37^ have reported the effect of the surfactant cetyltrimethylammonium bromide (CTAB) on alumina dispersion, weight fractions and properties of composite coatings. They obtained better alumina dispersion in the electrolyte and improved corrosion resistance properties when the concentration of CTAB was increased. Lin and Duh^38^ have investigated the effect of sodium dodecyl sulfate (SDS) on the surface roughness and wettability of Ni–P coatings. Chao and others studied the effect of cationic (CTAB) and anionic (SDS) surfactants on the properties of nickel carbon nanotube composite coatings and found that both cationic and anionic surfactants greatly influenced the mechanical and corrosion resistance properties of the prepared deposit.^39^ M. Rezaei-Sameti with group^40^ conducted a study on the effect of sodium dodecyl sulfate (SDS) and sodium saccharin on the surface morphology, microhardness and wear resistance of Cr-WC nanocomposite coatings. Their results indicated that as the content of WC was increased in the coatings, higher hardness and better wear resistance of the coatings were obtained with the increasing concentration of SDS in the electrolyte. Kartal *et al.*^22^ reported the effect of the surfactant SDS on the properties of Ni-WC composite coatings and found that the homogeneity of the WC dispersion increased and the mechanical and tribological properties of the composite coatings were improved significantly with the increasing concentration of SDS in the deposition process. A. Zarebidaki and S. R. Allahkaram^41^ investigated the influence of the SDS surfactant on the deposition behavior of Ni–P-CNT composite coatings. They demonstrated that the good dispersion and uniform distribution of CNTs in the coatings were achieved when the SDS concentration was increased in the electrolyte, and SDS was responsible for higher microhardness and better corrosion resistance of the coatings. Kyle Jiang and others^15^ observed that the addition of the anionic surfactant sodium dodecyl sulfate (SDS) increased the dispersion of graphene platelets in the deposition solution. An anionic surfactant (SDS) is mainly considered because it resists pin pores and assists the formation of metallic ion complexes.^42^

The use of an appropriate concentration ratio of surfactant and graphene in the electrolyte is highly significant in the preparation of nickel-graphene nanocomposite coatings. In this study, the effects of SDS at different concentrations in the deposition bath on the dispersion of graphene, surface morphology of the coatings, and weight percentage of the deposit are explored. Furthermore, the microstructure, mechanical properties and corrosion resistance behavior of the nanocomposite coatings affected by different surfactant concentrations in the electrolyte have been investigated.

## 2. Experimental

Natural graphite of 325 mesh was purchased from Qingdao Ruisheng Graphite Company Ltd. All other chemicals were of analytical grade and purchased from Beijing Chemical Works. The substrate material was Q235 carbon steel, and its chemical composition is provided in Table S1.†

### 2.1 Synthesis of graphene

Graphene oxide (GO) was synthesized by an improved Hummers method. Chemical exfoliation of graphite powder is a general approach to produce graphene oxide. In brief, 1.5 g graphite powder with 9 g KMnO_4_ was added gradually to a mixture of 180 mL H_2_SO_4_ and 20 mL H_3_PO_4_, and a slight exothermic reaction occurred 40 °C; then, the reaction mixture was heated to 55 °C and stirred for 12 h. After this, ice water (400 mL) along with 30% H_2_O_2_ (3 mL) was added to this mixture. The obtained mixture was kept at room temperature for 24 h for the purpose of sieving. The suspension was centrifuged and washed several times to decant the supernatants, and it was then dried at 60 °C in a vacuum desiccator for 24 h. Finally, the solid product GO was obtained.^43^ Graphene oxide was reduced into graphene by a chemical reduction method using the reducing agent hydrazine hydrate.^44^ The synthesized graphene was used in required concentrations in the electrodeposition process.

### 2.2 Electrochemical co-deposition process

The electrodeposition process was carried out to fabricate Ni/graphene nanocomposite coatings. The samples of carbon steel grade Q235 with a size of 20 × 10 × 2 mm were used as substrates (cathode) for deposition. Moreover, two plates of nickel (anode) with size 70 mm × 40 mm × 1 mm placed at both the ends of the bath solution served as the anode, and carbon steel serving as the cathode was adjusted between the two anode plates. Prior to electrodeposition, the samples were prepared by grinding different grades of abrasive emery papers (240#, 320#, 600# and 1200#) to obtain smooth and uniform surfaces. The substrate was first dipped in 10% HCL and then in a 5% H_2_SO_4_ solution to remove surface impurities, oil and oxide layers from the substrate. The basic bath composition and electrodeposition parameters are shown in Table 1. The properties of nanocomposite coatings obtained using the sodium dodecyl sulfate (SDS) surfactant at different concentrations of 0 g L^−1^, 0.2 g L^−1^ and 0.4 g L^−1^ in the electrolyte are characterized.

**Table 1:** Basic composition of the electrolyte and the electrodeposition parameters

Composition and condition (units)	Magnitude
NiSO_4_·6H_2_O (g L^−1^)	85–100
NiCl_2_·6H_2_O (g L^−1^)	12–15
H_3_BO_3_ (g L^−1^)	25–35
Graphene (g L^−1^)	0.2
pH	3–4
Temperature (°C)	45 ± 5 °C
Current density (A dm^−2^)	5
Time (min)	60
Stirring rate (rpm)	300

### 2.3 Characterization

Microhardness was determined using a Fischer HM2000 micro hardness tester. A load of 200 mN and a dwell time of 12 s were applied. The average hardness was obtained by testing five different positions of uniform distribution for each sample. The spectra for graphene were obtained using a Renishaw inVia RM1000 Raman microscope at a laser excitation wavelength of 532 nm. The adhesion strength of the nanocomposite coatings was measured using a DeFelsko (S/N AT05268CE) gauge meter. A Hitachi S-4700 scanning electron microscope was used for the observation of surface morphology, and an EDAX micro analyzer attached with a Hitachi S-4700 SEM was used to measure the elemental composition of nanocomposite coatings. The X-ray diffraction (XRD) patterns were obtained using a D/MAX-2500 X-ray diffractometer, with Cu Kα radiation (*λ* = 1.5406 Å). Bruker Multimode atomic force microscopy (AFM) was used to measure the surface roughness of the composite coatings.

Electrochemical impedance spectroscopic (EIS) measurements were performed using a PARSTAT 2273 electrochemical workstation (Princeton). The perturbation was 10 mV, and the applied scanning frequency range was 100 kHz to 10 mHz. The working electrode with a surface area of 1 cm^2^ was tested in a 3.5 wt% NaCl solution. The CS350 electrochemical workstation was used for potentiodynamic polarization curve measurements in a solution of 3.5 wt% NaCl. The surface area of the working electrode was about 1 cm^2^. A platinum plate was used as the counter electrode, and a saturated calomel electrode (SCE) was used as the reference electrode. The scanning rate was 0.5 mV.

## 3. Results and discussion

### 3.1 Characterization of graphene

**Fig. 1:**
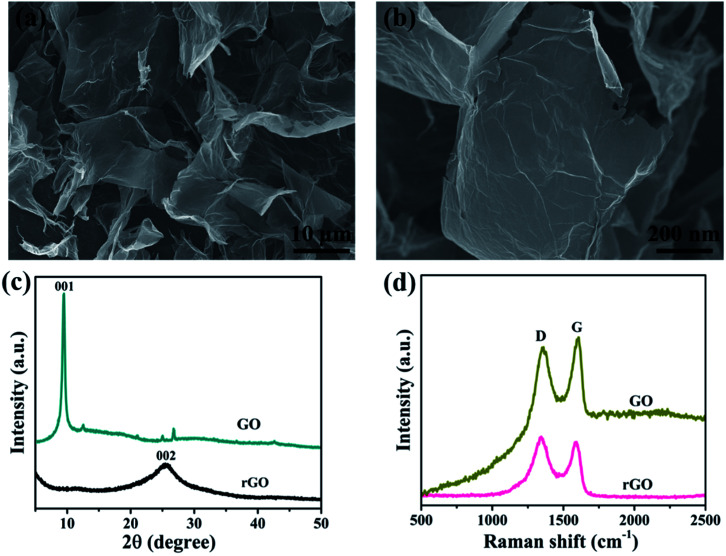
(a) and (b) SEM images of graphene, (c) X-ray diffraction patterns and (d) Raman spectra of graphene oxide and graphene.

**Fig. 2:**
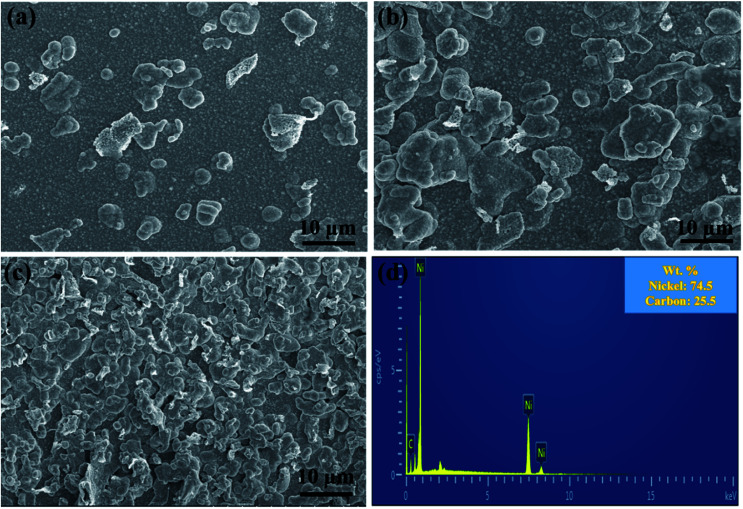
Surface morphologies of the Ni/graphene nanocomposite coatings fabricated using SDS at different concentrations: (a) 0 g L^−1^, (b) 0.2 g L^−1^ and (c) 0.4 g L^−1^, and (d) EDS of the deposit produced with 0.4 g L^−1^ SDS in the deposition bath.

**Fig. 3:**
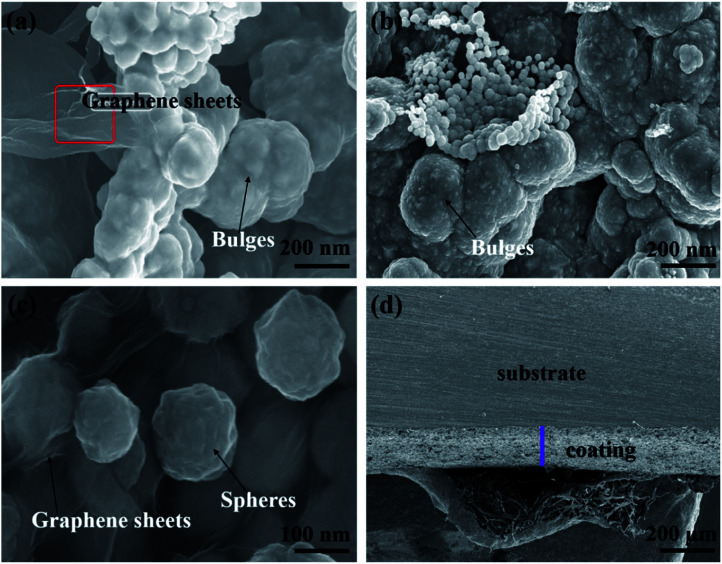
SEM images: (a) marked area indicates graphene sheets, (b) bulge morphology, (c) spherical growth and (d) cross-sectional view of the nickel-graphene nanocomposite coatings.

SEM images of graphene nanosheets are shown in Fig. 1. For contrast assessment of graphene and graphene oxide, Fig. 2 shows the X-ray diffraction patterns of (GO) and (rGO). The carbon peak (001) for GO sheets appeared at 10° corresponding to a definite *d* spacing of 0.8 nm as reported elsewhere.^43^ After reduction, (001) eventually disappeared, and a new peak (002) was observed at 2*θ* = 25.4° corresponding to an interlayer distance of about 0.4 nm, which was highly consistent with a previous report^43^ and indicated good arrangement of the interlayer distances of graphene. Furthermore, for confirmation, notable structural changes during the chemical reduction of GO are shown in Fig. 3. The Raman spectra of graphene showed that the D peaks appeared at ∼1350 cm^−1^ and G peaks appeared at ∼1590 cm^−1^, which ensured lattice distortions.^44^

### 3.2 Effect of SDS on the surface morphology and carbon content in nanocomposite coatings

Fig. 2 reveals the effect of different concentrations of SDS on the surface morphologies of nanocomposite coatings prepared from deposition bath containing 0.2 g L^−1^ graphene. The comparison analysis of the composite coatings obtained from electrolytes with SDS and without SDS and the effect of anionic surfactant on the surface morphologies are also demonstrated. It can be seen that the addition of the anionic surfactant SDS has increased the roughness of the composite coatings significantly.

The surface morphologies of composite coatings were observed to be much coarser when the surfactant SDS was added to the depositing electrolyte, as shown in Fig. S1,† and their roughness values for comparison are given in Table 2. It can also be observed that the roughness of the coatings is increased to a large extent when the surfactant SDS concentration increases to 0.4 g L^−1^ in the basic bath solution.

**Table 2:** Surface roughness of the nanocomposite coatings deposited at different SDS concentrations

SDS concentration (g L^−1^) in deposition bath	Roughness	
	Average roughness (*R*_a_), (nm)	Root mean square roughness (*R*_q_), (nm)
0	88.2	107.4
0.2	127	198
0.4	185	228

The SEM images show bulge morphologies (Fig. 3a and b) of nickel-graphene nanocomposite coatings fabricated at a SDS concentration of 0.4 g L^−1^ in the electrolyte. It can be seen perceptibly that when the SDS concentration is high in the deposition bath, large bulge morphology^45^ that comprises graphene nanosheets and nickel is observed in the coatings. The expected reason for the effect of the surfactant SDS can be explained as follows: during the electrodeposition process, nickel ions move and deposit on the cathode surface, and graphene sheets also tend to incorporate into a freshly grown nickel matrix. Moreover, the agglomeration of graphene sheets is increased, and bulge formation is observed due to the high conductivity^46^ and small size of graphene nanosheets because current density is higher around the graphene sheets than that in the other areas. Thus, the faster deposition of nickel ions would be around graphene sheets that would lead to the formation of higher bulge morphology in the composite coatings. Furthermore, a higher concentration of SDS facilitates better dispersion of the graphene sheets in the electrolyte, which is supportive for higher co-deposition of graphene sheets because the adsorption of surfactants on the graphene sheets causes electrostatic repulsion between graphene sheets that hinders the aggregation and promote fast and uniform deposition. In addition, some negative functional groups due to the presence of SDS in the electrolyte were likely to get adsorbed on the graphene sheets and raise the nickel ion deposition around the graphene sheets.

The cross-sectional image is shown in Fig. 3d, which is a good evidence for the efficiency of composite deposits, and the thickness of the nanocomposite coatings can be estimated. For further confirmation and evidence of SEM results, the effect of SDS on the surface morphology with a change in surface coarseness is provided in Table 2. The change in roughness with the increasing SDS concentration is also shown in Fig. S1.† The maximum roughness of the nickel-graphene nanocomposite is achieved with a 0.4 g L^−1^ SDS concentration in the deposition bath solution.

**Fig. 4:**
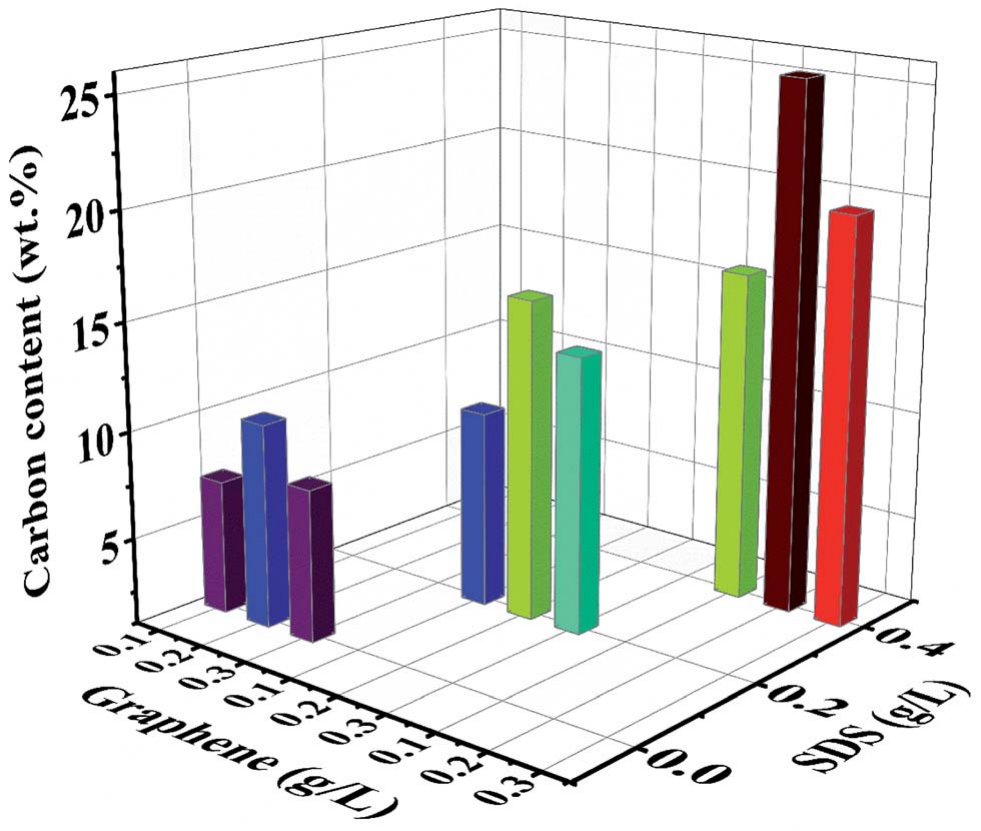
Effect of the surfactant (SDS) on the carbon content (wt%) of nickel-graphene nanocomposite coatings synthesized with different graphene concentrations in the bath solution.

EDS was performed to evaluate the composition of nanocomposite coatings. Fig. 4 shows the carbon content (wt%) in Ni/graphene nanocomposite coatings produced using different concentrations of SDS surfactants in the bath solution. It also demonstrates the composition of the composite coatings fabricated from the bath solution without surfactants. The results indicate that a better dispersion of graphene is obtained due to the presence of functional groups on graphene sheets that is responsible for the absorption of surfactants (SDS) on the surface of graphene platelets. This creates an electrostatic repulsion between graphene sheets and inclines to accelerate the carbon content in the produced coatings and promote the uniform distribution of graphene into the nickel matrix during the co-deposition process. Hence, a higher carbon content incorporated into the deposit is obtained when the surfactant concentration is increased.

### 3.3 Effect of SDS on the texture coefficients and grain sizes of composite coatings

**Fig. 5:**
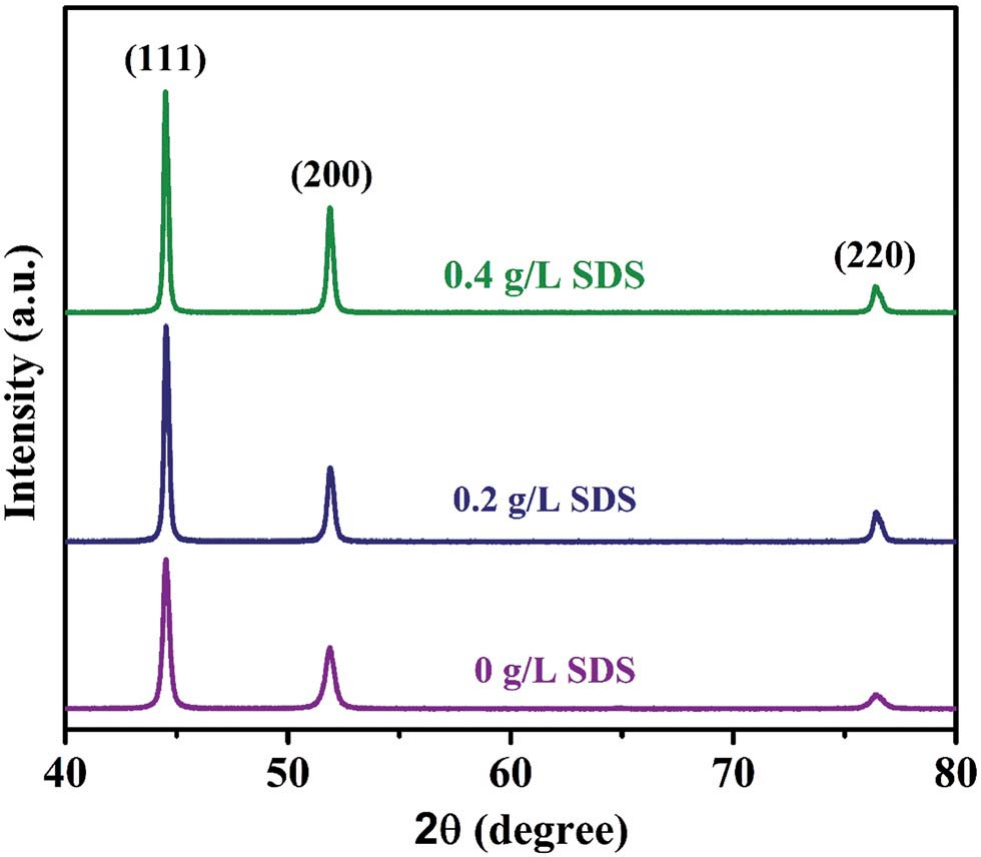
XRD patterns of nickel-graphene composite coatings prepared from the deposition bath containing different SDS concentrations.

X-ray diffraction patterns of the composite coatings obtained using different concentrations of SDS in the basic solutions are shown in Fig. 5. Huis and others ^47^ revealed the cluster size and the mean cluster size calculations, and they reported that the results calculated from XRD using the Scherrer's method and also TEM observations were relatively constant. Scherrer's formula is given below to calculate the average grain size of the composite coatings:^48^1
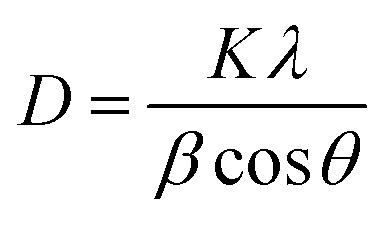
where *D* is the average crystalline size, *K* is the Scherrer constant, *β* is the FWHM, *θ* is the Bragg angle and *λ* is the wavelength with a value 0.15406 nm.

The preferred orientations were found by determining the texture coefficients using equation^15,49^ given as follows:2
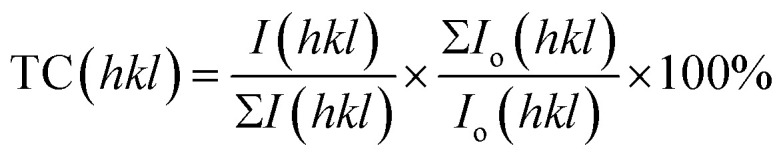
where *I*(*hkl*) is the reflection intensity of the tested samples and *I*_o_(*hkl*) is the refection intensity of the standard oriented sample.

Table S2† reveals the average grain sizes and texture coefficients of Ni-graphene nanocomposite coatings. The coating made with a higher percentage of surfactant SDS in the electrolyte shows reduced grain sizes, and it is undoubtedly seen that the coating produced from a higher SDS concentration in the deposition bath exhibits preferred orientations. Although it is observed that the plane (200) is the preferred orientation for the coating prepared without surfactants, with the increasing concentration of SDS in the electrolyte, there is an increasing trend of intensity for the preferred orientations at planes (111), (200) and (220). Zhang *et al.*^50^ reported that the coatings produced by face-centered cubic (fcc) metals showed a linear increase in the strain energy density of grains oriented at (*hkl*) with an angle between (100) and (*hkl*). In this observation, the analyzed results indicate a reduction in strain energy density with the increasing SDS concentration in the co-deposition process; this is mainly liable for the decrease in grain sizes and the relevant preferred orientations of the nickel-graphene nanocomposite coatings.

### 3.4 Effect of SDS on the mechanical and corrosion resistance properties

**Fig. 6:**
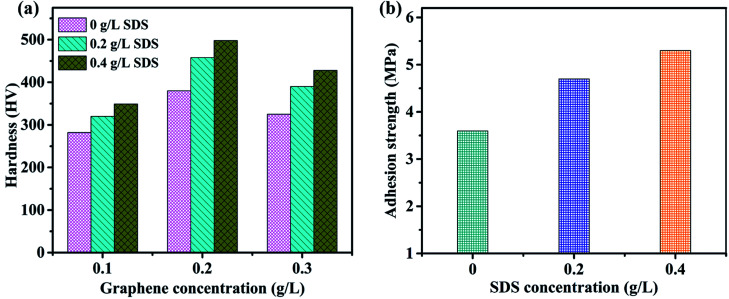
Effect of SDS on the microhardness (a) and adhesion strength (b) of composite coatings synthesized from the deposition bath containing different concentrations of SDS.

Adequate mechanical properties of nickel-graphene nanocomposites are greatly important to obtain superior corrosion resistance and other surface properties of materials. Fig. 6a illustrates the microhardness of nickel graphene composite coatings affected by different concentrations of SDS in the electrolyte containing 0.1 g L^−1^, 0.2 g L^−1^ and 0.3 g L^−1^ graphene sheets. The hardness of the obtained coatings prepared from the deposition bath containing SDS was increased when compared with that of the coatings produced without SDS in the electrolyte. This increasing tendency of microhardness of the composite despite higher concentration of SDS can be explained by the effect of surfactants on the deposition mechanism of reinforcement particles and metallic matrix of the nanocomposite; essentially, the adhesion strength between the matrix and the reinforcement particles plays an important role in the strengthening effect of any formed composite. It is expected that the surfactant is incorporated into the deposit after electrodeposition and can be supportive for the properties of the obtained coatings.^51^ In the present study, it may be justified that negative groups adsorb on the surfaces of graphene sheets, which are beneficial to increase the nickel ion attraction and develop a good combination between the nickel deposition layer and graphene sheets. Additionally, a higher carbon content in the composite is achieved as the SDS concentration reaches a maximum value to 0.4 g L^−1^, and this is also a vivid reason for the enhanced hardness and adhesive strength. The adhesion strength shown in Fig. 6b is also influenced *via* a similar trend as microhardness because the increased content of graphene sheets in the deposit due to higher SDS concentration in the electrolyte is largely liable for the tremendous mechanical properties of the fabricated nanocomposite coatings.

**Fig. 7:**
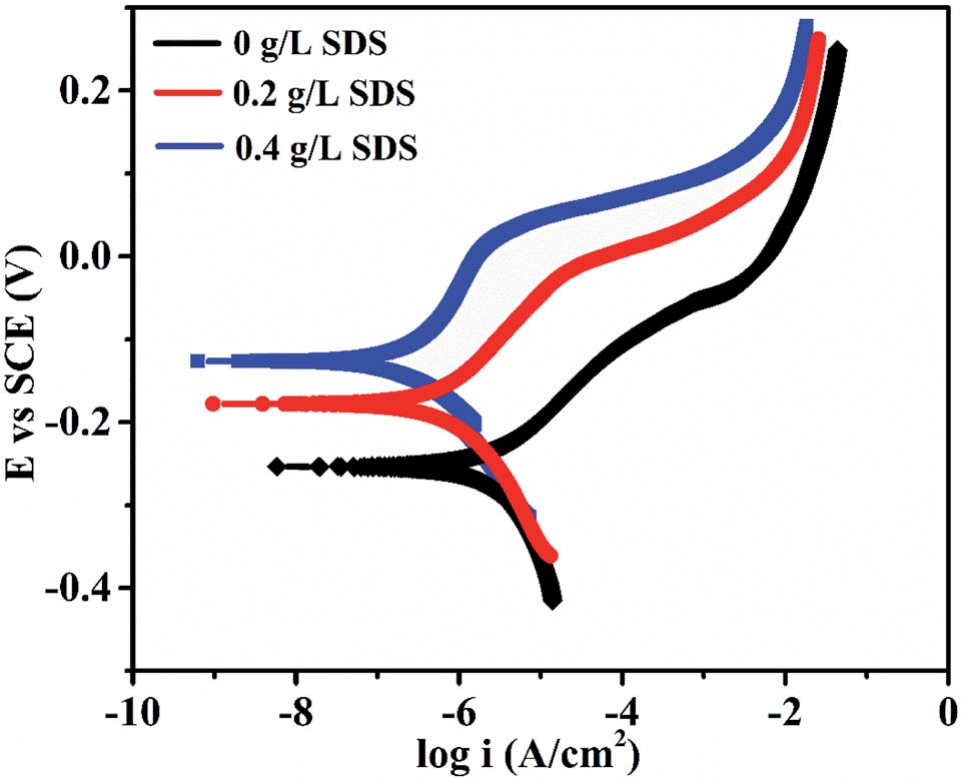
Effect of the surfactant SDS on the polarization curves of the composite coatings in a 3.5% NaCl solution.

**Fig. 8:**
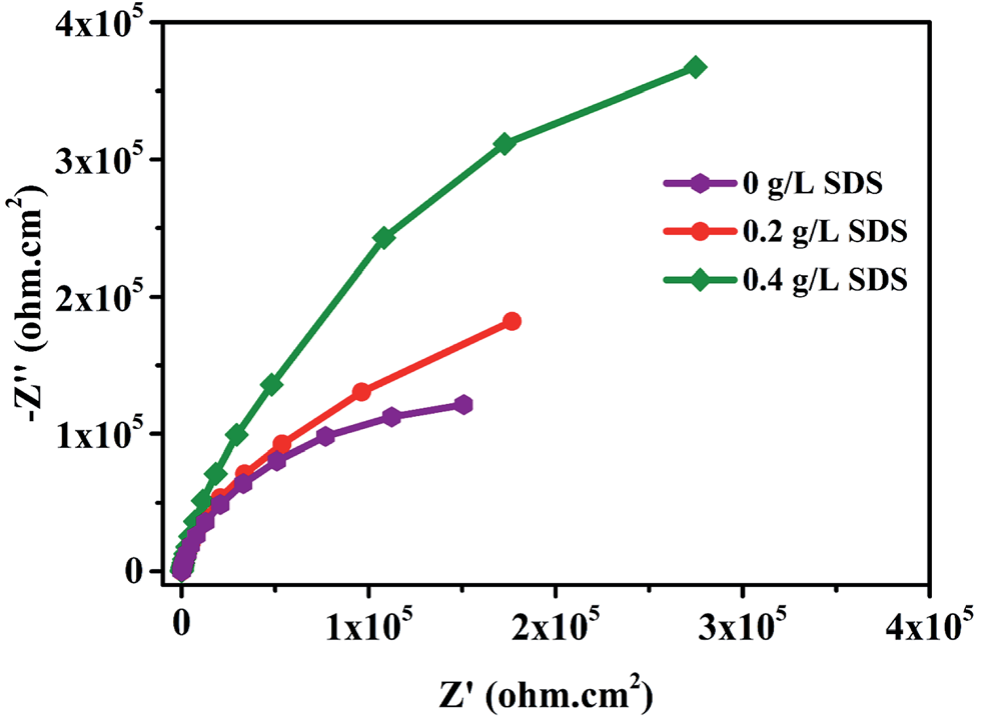
Effect of SDS on the impedance of composite coatings in a 3.5% NaCl solution.

The corrosion performance of nickel-graphene nanocomposite coatings is measured using polarization curves and electrochemical impedance spectroscopy in a 3.5% NaCl solution. Fig. 7 shows the potentiodynamic polarization curves to evaluate the corrosion behavior of the Ni/graphene nanocomposite coatings fabricated with different concentrations of SDS in the bath solution. The corrosion resistance of the composite coatings increased to some extent when the SDS concentration was increased in the deposition solution. Table 3 shows the corrosion current densities and corrosion potentials of the deposited coating at different concentrations of SDS in the electrolyte. Impedance measurements are shown in Fig. 8, and better corrosion resistance is observed at higher SDS concentrations in the electrolyte. Actually, the improved boundary adhesion strength of graphene sheets with the nickel matrix is expected to be achieved by the addition of the anionic surfactant SDS that would be responsible for the good electrochemical properties of the produced coatings. The corrosion resistance properties of the Ni/graphene composite coatings are better due to the reduced pinholes, voids and defective boundaries because of the optimum incorporation of SDS into the electrolyte during the deposition process.

**Table 3:** Effect of SDS concentration on the corrosion potentials and corrosion current densities of nickel-graphene nanocomposite coatings

Surfactant SDS (g L^−1^)	0	0.2	0.4
*I*_corr_ (A cm^−2^)	3.870 × 10^−6^	1.874 × 10^−6^	1.425 × 10^−6^
*E*_corr_ (V)	−0.253	−0.172	−0.129

Moreover, the superior corrosion resistance performance is due to the addition of graphene nanosheets that have tremendous mechanical properties and act as perpetual reinforcement in the nickel matrix. In fact, from the EIS results, we can evaluate the anti-corrosion property of coatings from the diameter of the semicircles, and the larger diameter ensures higher impedance to corrosion; we have found that the coating produced with 0.4 g L^−1^ SDS in the electrolyte has the highest impedance and possesses better corrosion resistance.

## 4. Conclusions

In summary, nickel-graphene nanocomposite coatings have been synthesized on carbon steel substrates from nickel sulfamate electrolyte containing graphene sheets with SDS surfactants at different concentrations of 0 g L^−1^, 0.2 g L^−1^ and 0.4 g L^−1^. The results indicate that the maximum carbon content and excellent mechanical properties of composite coatings are obtained due to higher concentration of anionic surfactant SDS in the electrolyte. A coarser surface morphology is observed owing to bulge formation on the surface of Ni/graphene nanocomposite coatings, and the average grain sizes are also decreased with the addition of SDS in the deposition process. Electrochemical tests demonstrate that the composite coatings exhibit more positive corrosion potentials (−0.129 V), lower corrosion current density (1.425 × 10^−6^ A cm^−2^) and higher impedance when 0.4 g L^−1^ concentration of SDS is present in the deposition bath that is responsible for the significantly superior corrosion resistance properties due to uniform distribution of graphene sheets in the nickel matrix during electrochemical co-deposition.

## Conflicts of interest

The authors declare that there is no competing financial interest.

## Supplementary Material

RA-008-C7RA13651J-s001
