# Supplementary material for: Effect of surfactant concentration in electrolyte on the fabrication and properties of nickel-graphene nanocomposite coating synthesized by electrochemical co-deposition
Source: RSC Adv. 2018 May 31;8(36):20039–47. doi: 10.1039/c7ra13651j (PMC9080783; doi:10.1039/c7ra13651j)
Supplement: RA-008-C7RA13651J-s001 [file RA-008-C7RA13651J-s001.pdf]

Electronic Supplementary Material (ESI) for RSC Advances.
This journal is © The Royal Society of Chemistry 2018

Electronic Supplementary Information (ESI)

**Effect of surfactant concentration in electrolyte on the fabrication and properties of nickel-graphene nanocomposite coatings synthesized by electrochemical co-deposition**

Ghulam Yasin[a,b*], Muhammad Arif[a], Muhammad Naeem Nizam[a], Muhammad Shakeel[a], Muhammad Abubaker Khan[c], Waheed Qamar Khan[d], Tahira Mehtab Hassan[e], Zaheer Abbas[a], Iman Farahbakhsh[f] and Yu Zuo[a*]

[a] State Key Laboratory of Chemical Resource Engineering, Beijing University of Chemical Technology, Beijing 100029, P.R. China
[b] BUCT-CWRU Joint International Laboratory, State Key Laboratory of Organic-Inorganic Composites, Center for Soft Matter Science and Engineering, College of Energy, Beijing University of Chemical Technology, Beijing 100029, P.R. China
[c] College of Materials Science and Engineering, Beijing Institute of Technology, Beijing 100081, P.R. China
[d] School of Materials Science and Engineering, Beijing University of Technology, Beijing 100029, P.R. China
[e] Department of Physics, The Islamia University of Bahawalpur, Bahawalpur, 63100, Pakistan
[f] Department of Engineering, Quchan Branch, Islamic Azad University, Quchan, Iran

**Corresponding Authors**

* Email: zuoy@mail.buct.edu.cn (Z.Yu)

* Email: yasin@mail.buct.edu.cn (G.Yasin)

**ORCID**

Ghulam Yasin: 0000-0001-8794-3965

**Table S1.** Chemical composition (wt.%) of carbon steel grade (Q235).

| **Elements** | **C** | **Si** | **Mn** | **S** | **P** | **O** | **N** | **Fe** |
|---|---|---|---|---|---|---|---|---|
| Wt.% | 0.14-0.20 | 0.30 | 0.30-0.70 | <0.04 | <0.04 | 0.01 | 0.004 | bal. |

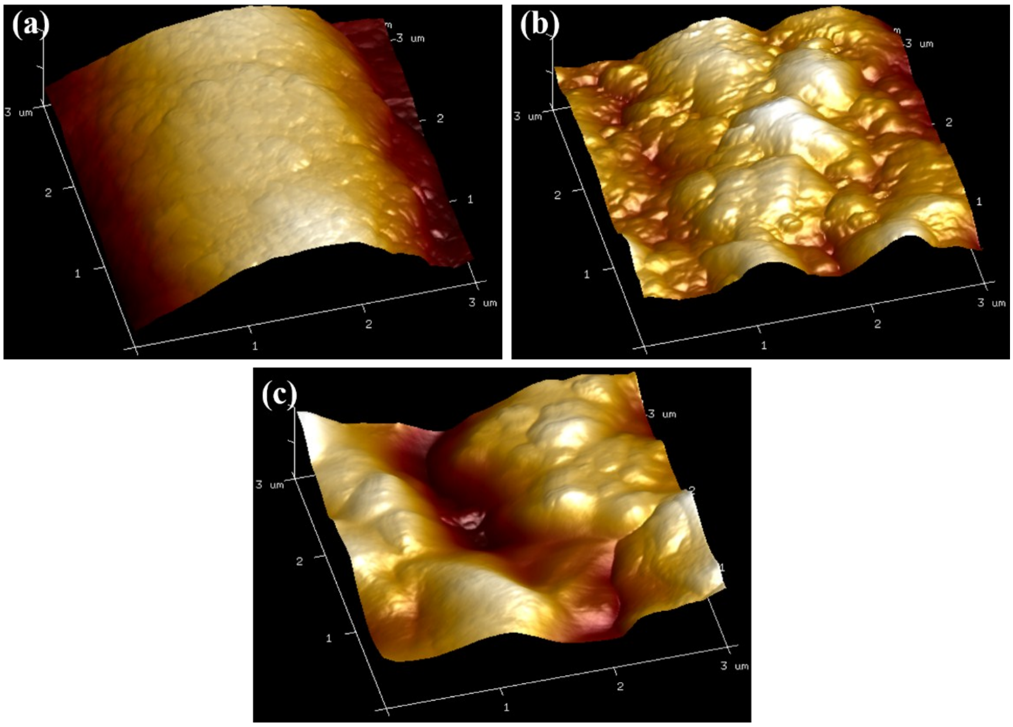


**Figure S1.** AFM micrographs of Ni/graphene nanocomposite coatings produced at different concentrations of SDS: (a) 0 g/L, (b) 0.2 g/L and (c) 0.4 g/L in the deposition solution.

**Table S2.** Effect of SDS on the grain sizes and texture coefficients of Ni/graphene coatings.

| **SDS** | **Grain sizes** | **Texture coefficients** | | |
|---|---|---|---|---|
| (g/L) | (nm) | TC(111) | TC(200) | TC(220) |
| 0 | 26.59 | 0.36 | 0.57 | 0.07 |
| 0.2 | 23.07 | 0.24 | 0.66 | 0.10 |
| 0.4 | 19.2 | 0.18 | 0.71 | 0.11 |
